# Physiological epicotyl dormancy and its alleviation in seeds of *Yunnanopilia longistaminea*: the first report of physiological epicotyl dormancy in China

**DOI:** 10.7717/peerj.3435

**Published:** 2017-07-19

**Authors:** Guan-song Yang, Liu Yang, Yue-hua Wang, Shi-kang Shen

**Affiliations:** School of Life Sciences, Yunnan University, Kunming, Yunnan, the People’s Republic of China

**Keywords:** Radicle emergence, Epicotyl dormancy, Endangered plant, Stratification, *Yunnanopilia longistaminea*

## Abstract

*Yunnanopilia longistaminea* is an endangered monotypic species belonging to Opiliaceae. This edible plant is an important germplasm source with a high economic value in China if propagation were less difficult. Seed dormancy and germination of this species were investigated to improve propagation. Considering seeds have a fully developed embryo and mature and are dispersed in summer, and radicles and epicotyls emerge the following autumn and next spring, respectively, we hypothesized that *Y. longistaminea* seeds may undergo physiological epicotyl dormancy. Seed moisture content and viability decreased as dehydration occurred. Thus, the seeds may be recalcitrant. The seed germination of this species involves two stages: radicle emergence and epicotyl (shoot) emergence. The optimum temperature was 28 °C and 28 °C/20 °C to radicle emergence. The optimum GA_3_ solution for the seeds undergoing shoot emergence was 100 mg L^−1^. The percentages of shoot emergence in seven and 14 days stratification at 5 °C were slightly higher than those in other groups. This study is the first to describe physiological epicotyl dormancy in *Y. longistaminea* seeds. Under natural conditions, seeds are subjected to *Y. longistaminea* a autumn → winter → spring temperature. Warm moist conditions and cold stratification can improve radicle emergence and alleviate epicotyl dormancy, respectively. The duration of cold stratification also significantly affects the epicotyl dormancy release of *Y. longistaminea*. Optimal dormancy breakage methods are warm (28 °C/20 °C) → cold (5 °C) → GA_3_(100 mg L^−1^) → warm (28 °C/20 °C).

## Introduction

Seed dormancy is a survival mechanism through which the timing of germination and distribution area are adjusted on the basis of different environmental conditions (Copete et al., 2011). Temperate plant seeds are typically dormant by the time of dispersal maturity; as such, these seeds require an appropriate temperature to germinate and for the seedlings to grow (Baskin & Baskin, 2004; Baskin, Baskin & Li, 2000). Dormancy ensures seed germination at appropriate temperature or rainfall conditions; this phenomenon also results in a high probability of successful seedling establishment (Bewley, Black & Halmer, 2006).

*Yunnanopilia longistaminea* (WZ Li) CY Wu et DZ Li, a monotypic species belonging to Opiliaceae, is a vulnerable plant endemic to Red River Valley, Yunnan Province, Southwest China. As the sole member of a geographically isolated genus, *Y. longistaminea* plays a significant part in the phylogeny and evolution of Opiliaceae (Wu & Li, 2000). Furthermore, *Y. longistaminea* is an important resource plant consumed as a delicious wild woody vegetable. However, this species is threatened because of excess utilization and habitat destruction. As a key component of germplasm conservation and access to wild plant resource, artificial propagation, including seed germination and seedling establishment, is an essential step in conservation procedure plans (Shen et al., 2015; Khanna et al., 2013). However, no study has been conducted on the germination characteristics of *Y. longistaminea* seed.

**Figure 1:**
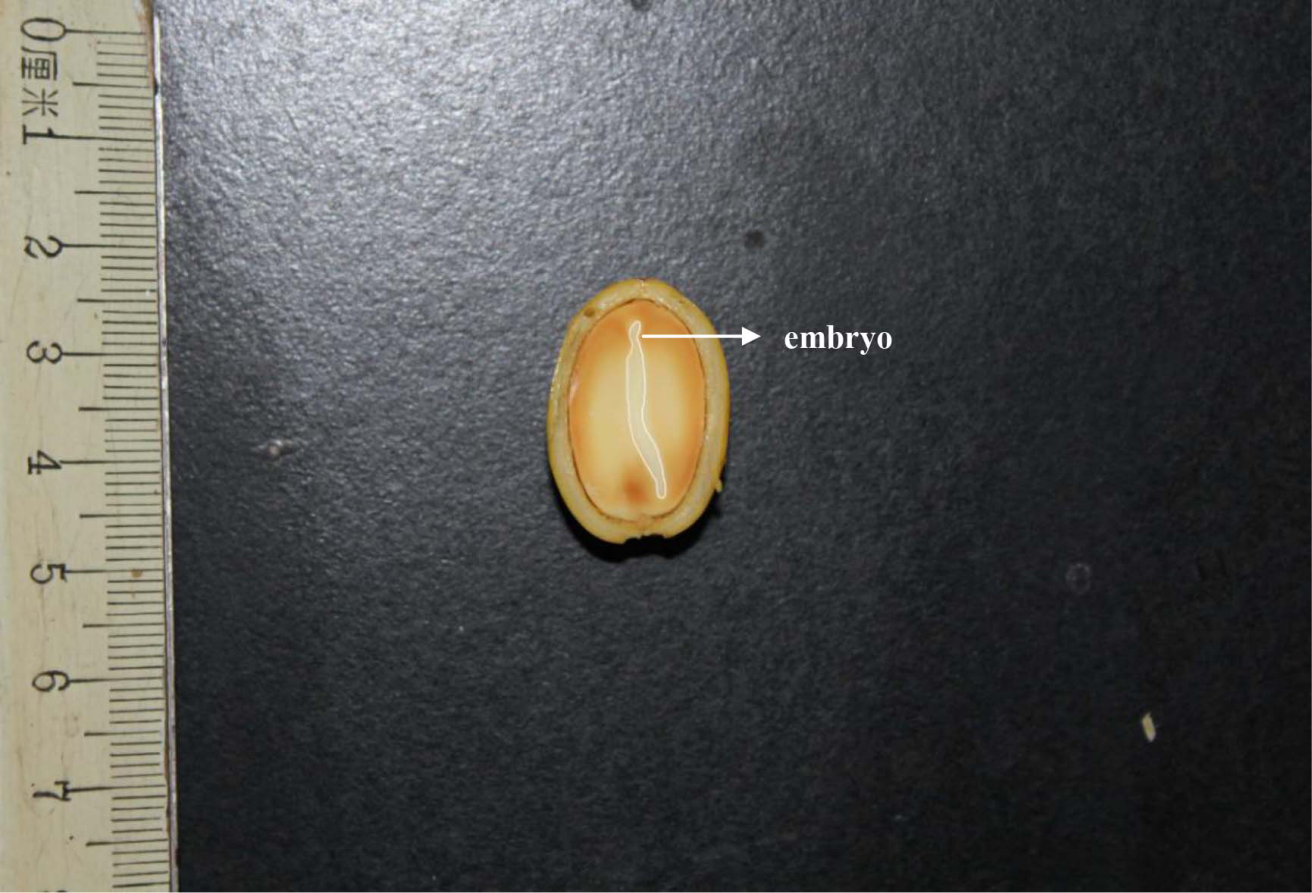
Characteristics of *Y. longistaminea* seeds.

With regard to the desiccation tolerance and storage behavior, seeds are always divided into two categories; orthodox or recalcitrant (Daws et al., 2004; Daws, Garwood & Pritchard, 2006). Recalcitrant seeds are intolerant dehydration and must avoid the dry and low temperature storage (McCartan, Jinks & Barsoum, 2015). To the best of our knowledge, limited information on seed characteristic and germination in Opiliaceae has been published. Willis, Baskin & Baskin (2014) presented the phylogeny of seed plant families with current dormancy classes and proposed that Opiliaceae seeds always exhibit morphophysiological dormancy (MPD). Morphophysiological dormancy (MPD) is the seeds with underdeveloped embryos and requires a dormancy-breaking treatment such as a specific temperature cue (Adams, Baskin & Baskin, 2003; Baskin & Baskin, 2003; Baskin & Baskin, 2004; Santiago, Ferrandis & Herranz, 2015). Epicotyl dormancy is a type of dormancy that the radicle develops into roots and the hypocotyls do not elongate, and shoot emergence always delay after radicle protrusion (Baskin & Baskin, 2004; Kondo et al., 2004; Fenner & Thompson, 2005; Hidayati, Baskin & Baskin, 2005). Two types of epicotyl dormancy include physiological epicotyl dormancy and morphophysiological epicotyl dormancy thus far reported in many species growing in various vegetation types (Baskin & Baskin, 1998; Barton, 1933; Nikolaeva, 1977; Jayasuriya et al., 2012; Santiago, Ferrandis & Herranz, 2015). In natural conditions, the seeds of *Y. longistaminea* mature and are dispersed in summer, and radicle emergence and cotyledon emergence occur in the following autumn and next spring, respectively. That is to say, there is a significant delay between radicle emergence and cotyledons emergence during germination. However, seeds of *Y. longistaminea* have a fully developed embryo (Fig. 1). Thus, we hypothesized that *Y. longistaminea* seeds may undergo physiological epicotyl dormancy. This study generally aimed to characterize the germination requirements for radicle and epicotyl (shoot) emergence of *Y. longistaminea*. The seed desiccation tolerance was also examined because large seeds are always sensitive to dehydration.

## Materials and Methods

### Ethics statement

The seeds of *Y. longistaminea* collected in this study, we got the field permission of this study from the Wildlife Protection and Administration Office under the Forestry Department of Yunnan, China. Our sampling was not affected the regular growth of *Y. longistaminea*, and it was solely used for scientific research. This study was financially supported by grant 31360074 from the National Science Foundation of China.

### Sample site and seed collection

*Y. longistaminea* is mainly distributed in the Red River Valley in Yunnan Province, Southwest China. All individual plants were distributed vertically from 1,000 m to 1,400 m in elevation. The minimum temperature in this region was 5.2 °C in January and the maximum temperature was 35.8 °C in July. The annual mean temperature was 18 °C. The annual average rainfall was approximately 2,000 mm, which is mostly recorded between June and September. The region is characterized by a typical high humid geographical environment with an annual mean relative humidity 80–90%.

Drupes of *Y. longistaminea* mature between June and July. In this study, mature fruits of *Y. longistaminea* were collected manually for germination tests in 2015. *Y. longistaminea* drupes got a yellow tone on the skin when they were fully matured. The seeds were manually separated from the drupes and divided into two portions. One portion was reserved in a sealed polyethylene bag that avoid seed dehydration for germination tests; the other portion was immediately placed under moisture-controlled condition for desiccation tolerance tests. All non germinated seeds were dead in our experiments.

### Seed morphology, weight, and moisture content

The dimensions (breadth and length) of the seeds were measured using a vernier caliper in triplicates. The seeds were measured with three replicates of 10 seeds each, and the 1,000-seed weight was determined. Moisture content was examined gravimetrically at 103 °C ± 2 ° C for 17 h in accordance with standard procedures (ISTA, 2017). The final moisture content was expressed as dry weight basis (%) (Shen, Wang & Ma, 2010). Three replicates of 10 seeds each were used to determine moisture content expressed as dry mass basis.

### Effect of temperature and desiccation on radicle-emergence

#### Temperature

Seeds were surface-sterilized with 0.85% sodium hypochlorite for 1 min and washed with distilled water. Fresh seeds were placed on moist autoclaved fine sand in a plastic box (25 × 10 × 8 cm length, width, height). The seeds were incubated within a 12 h/12 h photoperiod at daily alternating temperatures of 28 °C/15 °C and 28 °C/20 °C and at constant temperatures of 5 °C, 15 °C, 20 °C, 25 °C, 28 °C and 30 °C. All temperature treatments were set in four replicates with 10 seeds in each replicate. The seeds were then checked for radicle emergence every five days throughout each treatment duration after seven days. Distilled water was added as needed to ensure that moisture was non-limiting during germination.

#### Desiccation

The seeds were dried for 0, 6, 9, 12, 24 and 36 h in an airtight glass desiccator overlaid with activated silica gel (silica gel:seeds > 10:1). The moisture content of the seeds was determined in weight in each drying treatment. Moisture content was examined gravimetrically at 103 °C ± 2 °C for 17 h in accordance with standard procedures (ISTA, 2017). Afterward, four replicates of 10 seeds each of dried seeds were tested for germination at room temperature (18/25 °C) in daylight for 12 h. The seeds were then checked for radicle emergence every 5 days throughout each treatment duration.

### Effect of GA_3_ treatment and cold stratification on the epicotyl dormancy release of radicle-emerged seeds

#### GA_3_

This experiment aimed to determine whether GA_3_ eliminates the epicotyl dormancy of radicle-emerged *Y. longistaminea* seeds. All seeds keep under near-natural temperature conditions to grow. The seeds were randomized in experiment. Four replicates of 10 seeds each of seeds with the same radicle lengths (about 5 cm) were independently soaked in 0, 50, 100 and 200 mg L^−1^ GA_3_ (95–100% purity; Sigma-Aldrich, St. Louis, MO, USA) for 10 h at 28 °C/20 °C in daylight for 12 hr and incubated to under the same conditions to test the epicotyl plumule emergence.

#### Cold stratification

The seeds with similar radicle lengths (about 5 cm with 60 days growth after radicle emergence) were stratified at 5 °C. During stratification four replicates of 10 seeds were removed after 0, 7, 14, 21 and 35 days and monitored for epicotyl-plumule emergence. The seeds with observed epicotyl-plumule emergence were transferred into a temperature and light-controlled incubator (BSG-400 Illuminating Incubators; Shanghai, China) and incubated at an alternating temperature of 28 °C/20 °C in the plastic boxes. Epicotyl dormancy was considered broken when the cotyledons emerged partly out of the seed coat (Hidayati, Baskin & Baskin, 2005).

#### Statistical analysis

In the present study, radicles grown to ≥2 mm, shoot grown to ≥5 mm were considered as radicle-emergence and epicotyl-plumule emergence criterion, respectively (Liu et al., 2005). Taking observations was halted when the surplus seeds (no germinated seeds) were for a consecutive one month until eventually decayed.

The shoot emergence percentage (SEP) and the final radicle emergence percentage (REP) were determined as follows:

\begin{eqnarray*}\text{Radicle emergence percentage} (\text{%})= \left( \frac{\text{number of radicle emerged seeds}}{\text{number of seeds per sample}} \right) \times 100 \end{eqnarray*}

\begin{eqnarray*}\text{Shoot emergence percentage} (\text{%})= \left( \frac{\text{number of shoot emerged seeds}}{\text{number of radicle has emerged}} \right) \times 100. \end{eqnarray*}

Arcsine transformation was applied for REP and SEP data before statistical analysis was conducted to ensure variance homogeneity. Data given in figures were not transformed. The factors influencing REP and SEP were analyzed through ANOVA. If a significant difference was detected by ANOVA, Fisher’s least significant difference (LSD) was applied to accomplish multiple comparison tests. Statistical analyses were performed in SPSS 18.0.1 (SPSS Inc., Chicago, Illinois, USA). The critical level of significance was *P* = 0.05 in all the tests.

## Results

### Characteristics of *Yunnanopilia longistaminea* seeds

*Y. longistaminea* seeds generally consist of a seed coat, an embryo and an endosperm. The fresh seeds were 19.12 ± 1.22 mm (average ± SE, *n* = 10) in length and 12.68 ± 0.63 mm (average ± SE, *n* = 10) in width. The 1,000-fresh seed weight was 2,580 ± 108 g. There were two stages in *Y. longistaminea* of seedling development, the first stage was radicle-emergence; the second stage was shoot emergence (epicotyl-plumule emergence).

### Effects of temperature and desiccation on radicle emergence

#### Temeperature

**Figure 2:**
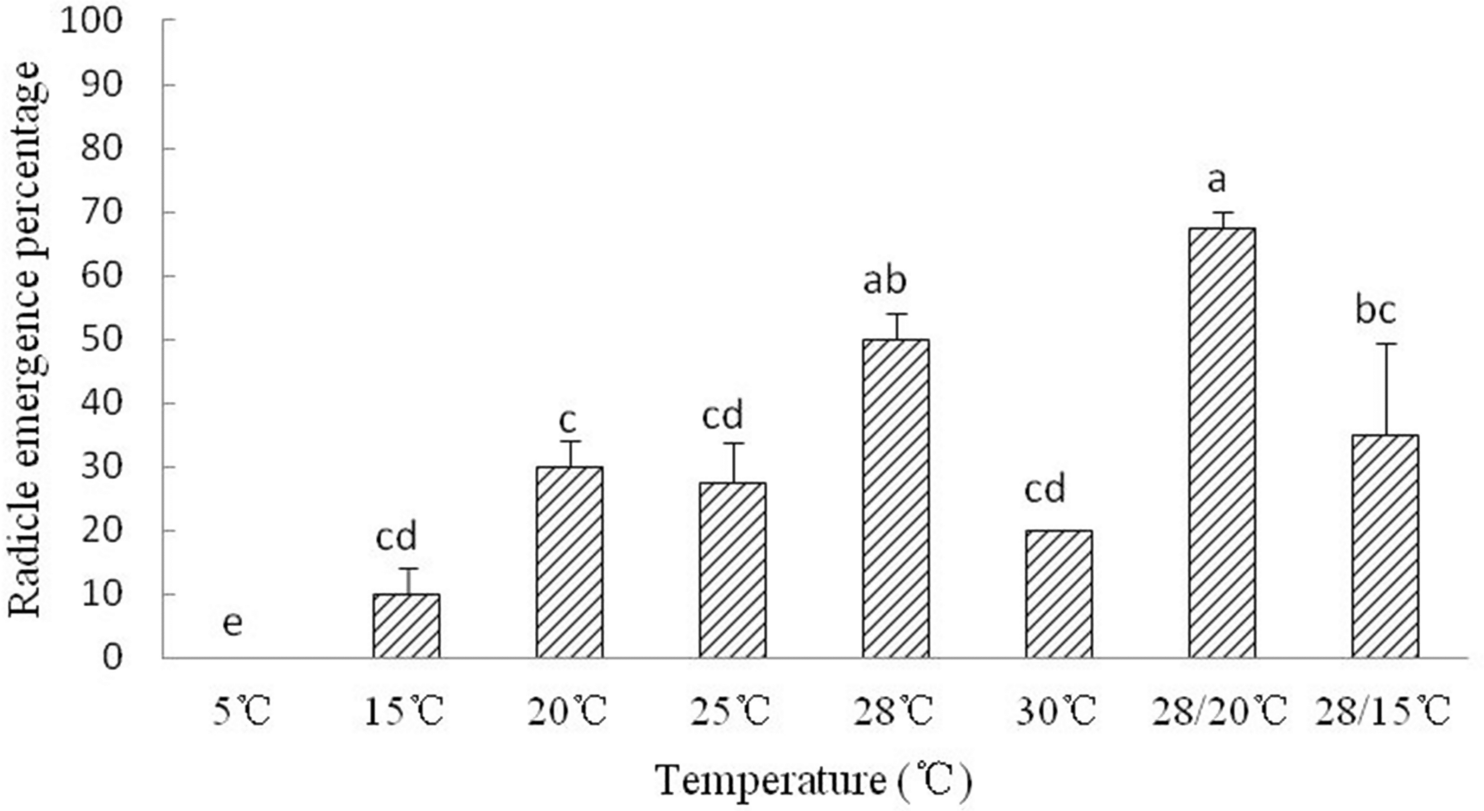
Figure of radicle emergence of *Y. longistaminea* at different temperatures. Seed radicle emergence of *Y. longistaminea* at different temperatures and a photoperiod of 12/12 h day/night. Values are mean ± standard error (*n* = 3). Different letters indicate significant differences between the temperatures (*P* < 0.05).

The radicle emergence of *Y. longistaminea* seeds is sensitive to temperature. Radicle emergence in *Y. longistaminea* seeds occurred about 8–10 days. Radicles did not emerge from any of the newly matured seeds kept at 5 °C. The optimum temperature for radicle emergence were 28 °C and 28 °C/20 °C. High and low temperature significantly decreased the REP (Fig. 2).

#### Desiccation treatments

**Figure 3:**
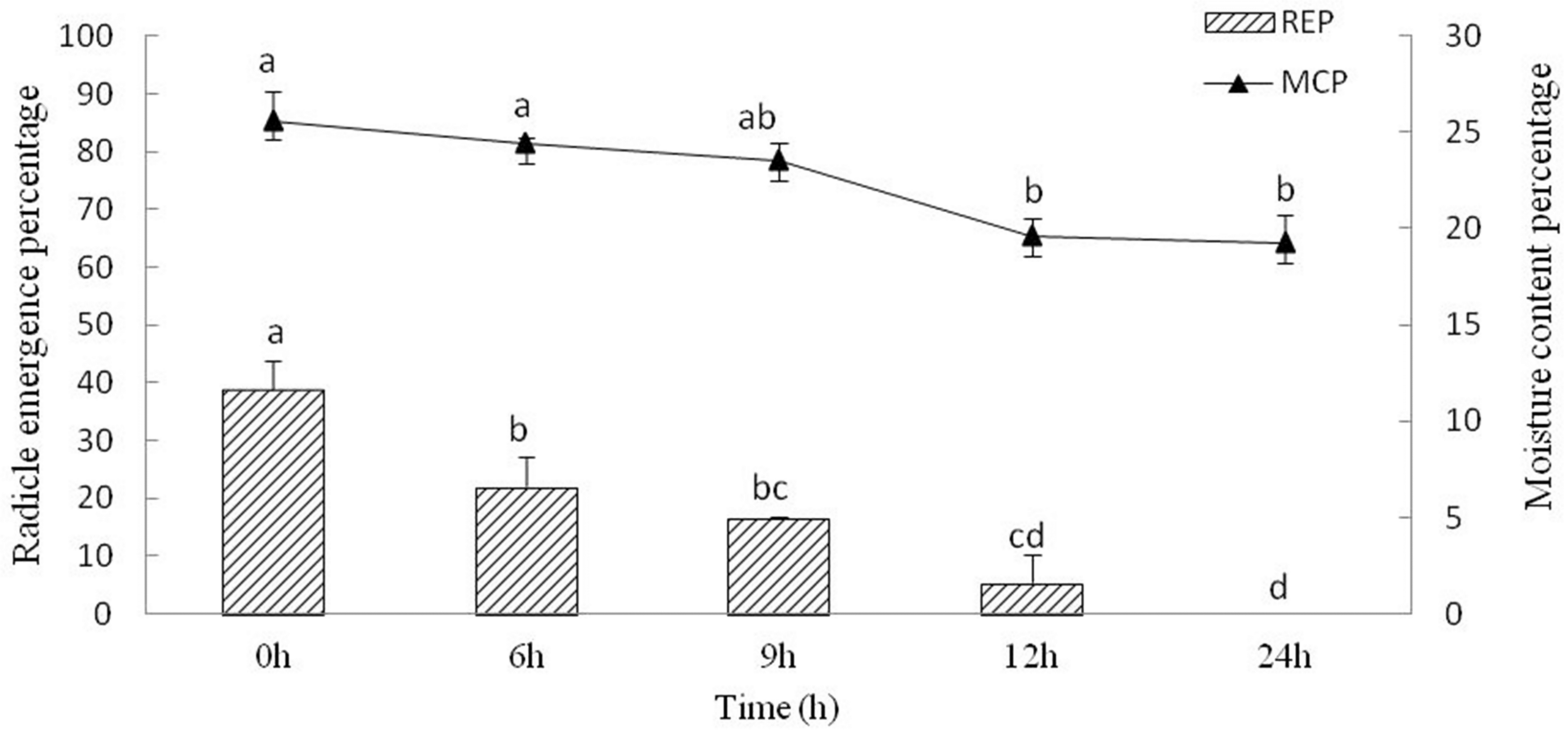
Seed radicle emergence of *Y. longistaminea* at different moisture content and dehydration time. Values are mean ± standard error (*n* = 3). Different letters indicate significant differences between the dehydration time (*P* < 0.05).

The moisture content of *Y. longistaminea* seeds steadily decreased for 24 h of drying, and 19.2 ± 1.5% moisture was reached after 24 h of dehydration. The REP of the seeds decreased during dehydration. The REP decreased from 38.9 ± 4.8% to 0 when the seed moisture content decreased from 25.6 ± 1.6% to 19.2 ± 1.5% (Fig. 3). It indicated that REP is sensitive to dehydration.

### Effect of GA_3_ treatment and cold stratification on the epicotyl dormancy release of radicle-emerged seeds

#### GA_3_

**Figure 4:**
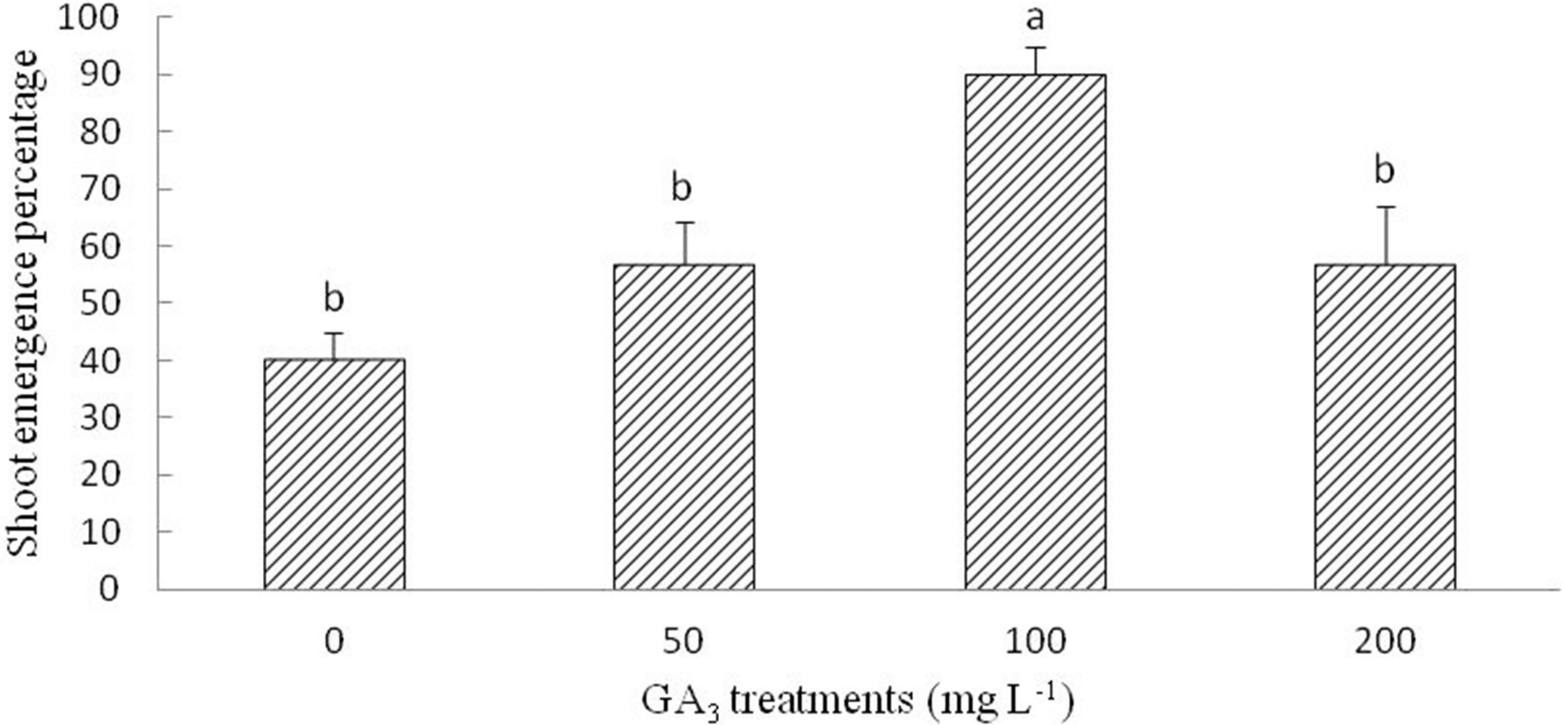
Figure of gibberellic acid (GA_3_) treatment on shoot growth of *Y. longistaminea*. Effect of gibberellic acid (GA_3_) treatment with shoot growth of *Y. longistaminea* under a 12/12 h photoperiod and 28/20 °C. Values are mean ± standard error (*n* = 3). Different letters indicate significant differences between the different GA_3_ solutions (*P* < 0.05).

The optimum GA_3_ solution for the shoot emergence of radicle-emerged seeds was 100 mg L^−1^. Although multiple range analysis (LSD) did not show significantly differences at 50, 200, and 0 mg L^−1^ (control) GA_3_ treatments, the SEP of the seeds with emerged radicles was improved by the GA_3_ treatment (Fig. 4).

#### Cold stratification

**Figure 5:**
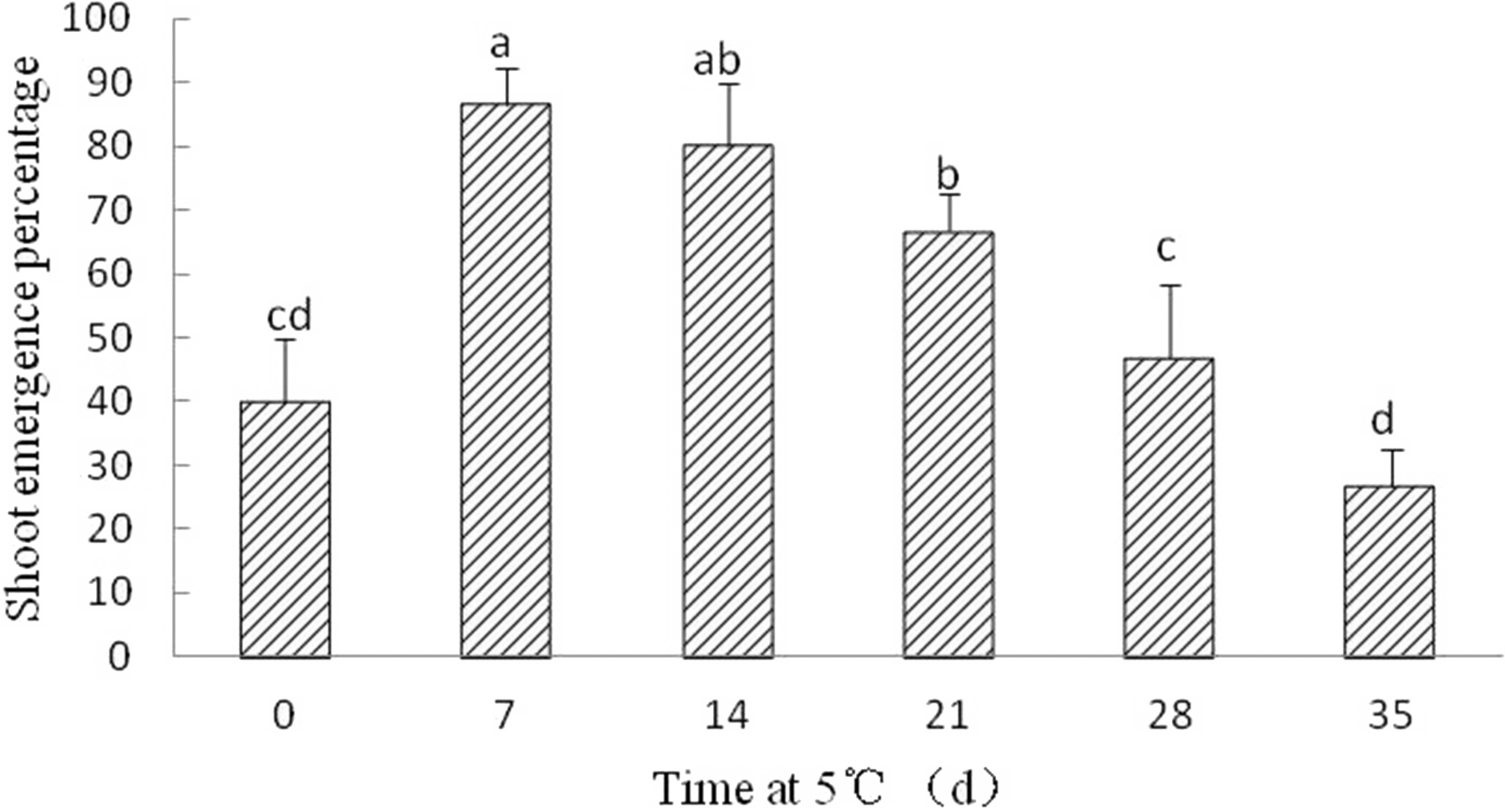
Figure of cold treatment on shoot growth in germinated seeds of *Y. longistaminea*. Effect of shoot growth in germinated seeds of *Y. longistaminea* following 0–35 days of cold treatment at 5 °C under a 12/12 h photoperiod and 28/20 °C. Values are mean ± standard error (*n* = 3). Different letters indicate significant differences between the different cold treatment (*P* < 0.05).

Although radicle emergence occurred with 8–10 days, epicotyl emergence was delayed at least 60 days in *Y. longistaminea* seeds without cold stratification after radicle protrusion. After seven, 14, and 21 days of cold stratification at 5 °C, the shoot emergence was significantly increased to 86.67 ± 5.77%, 80 ± 10%, and 66.67 ± 5.77%, respectively. By contrast, SEP declined when the duration of cold stratification was prolonged (Fig. 5).

## Discussion

Seed of *Y. longistaminea* has a fully developed embryo (embryo axis plus cotyledons) that nearly fills the whole seed (Fig. 1). According to the seed dormancy classification, seeds with morphological dormancy (MD) and morphophysiological dormancy (MPD) always have an underdeveloped embryo that needs time to grow (the dormancy period) within the seed before the radicle emerges (Baskin & Baskin, 1998; Baskin & Baskin, 2004; Baskin & Baskin, 2004; Santiago, Ferrandis & Herranz, 2015). We therefore think that seeds of *Y. longistaminea* can not have either MD or MPD. However, seed germination of *Y. longistaminea* involves distinct two stages: radicle and epicotyl emergence. The drupes of *Y. longistaminea* matured and dispersed between June and July, and seedlings emerged in spring next year under natural conditions, within is about 10 months. In this case, they were exposed to a sufficient warm period after they were dispersed for radicle emergence. They also received cold stratification in winter for epicotyl emergence. In addition, epicotyl emergence occurred at least 60 days after radicle emergence under laboratory conditions. This delay in shoot emergence, could be considered as a type of epicotyl dormancy according to the epicotyl dormancy definitions. This phenomenon is consistent with that of Jayasuriya et al. (2010), who reported that seeds of *Humboldtia laurifolia* have physiological epicotyl dormancy, a subclass of physiological dormancy (Jayasuriya et al., 2012). Other species in the Clusiaceae, Fabaceae, Fagaceae, Lecythidaceae, and Oleaceae families were also experienced this dormancy type (Allen & Farmer, 1977; Baskin & Baskin, 1998; Chien et al., 2004; Agyili et al., 2007; Jayasuriya et al., 2010). The present study appears to be the first report of a species in China with physiological epicotyl dormancy. Furthermore, the fact that approx. 40% of the radicle-emerged seeds have epicotyl emergence without cold stratification and GA_3_ effectively improved epicotyl emergence suggests that the physiological epicotyl dormancy involved here is non-deep level (Baskin & Baskin, 1998; Baskin & Baskin, 2004). Although a short period of cold stratification (7 and 14 days) significantly improved epicotyl emergence, approx. 40% of the radicle-emerged seeds have epicotyl emergence without cold stratification indicated that cold stratification may be not essential for its shoot emergency.

Dehydration-sensitive seeds are typically large and they mature during the rainy season. The species producing dehydration sensitive seeds mostly occur in tropical subtropical forests (Pammenter & Berjak, 2000; Shen et al., 2015). In the present study, *Y. longistaminea* seeds matured in the rainy season (June) at >25 °C in their natural habitat. In the dehydration test, the seed moisture content of *Y. longistaminea* decreased gradually during desiccation. Seed viability also declined as the duration of dehydration was prolonged. The viability was almost zero at about 19% seed moisture. Thus, we conclude that *Y. longistaminea* seeds are recalcitrant. In general, recalcitrant seeds have a rapid germination strategy (both radicle and shoot emergence) that allows them to escape adverse environments (Pammenter & Berjak, 2000; Shen & Wang, 2009; Shen, Wang & Ma, 2010). However, it is interesting that seeds of almost all the species reported as having a fully developed embryo and epicotyl dormancy are recalcitrant (Jayasuriya et al., 2012). Jayasuriya et al. (2012) hypothesized that early radicle emergence is a germination strategy for the recalcitrant seeds to maintain viability. Our present observation of *Y. longistaminea* seeds supported this hypothesis. However, the ecological significance of physiological epicotyl dormancy in recalcitrant seeds still need further elucidation.

In summary, this study is the first to demonstrate that *Y. longistaminea* seeds undergo epicotyl dormancy, it exhibit recalcitrance and storage behavior. Considering the high frequency of human disturbances in the natural habitat of this endangered species, we speculate that the prolonged duration of *Y. longistaminea* seed germination may affect its natural regeneration. Thus, we recommend the artificial promotion of dormancy release and germination for its conservation and management.

##  Supplemental Information

10.7717/peerj.3435/supp-1 Data S1 Raw data exported from different treatments (temperature, moisture content, GA_3_, cold treatment) on germination of *Y. longistaminea* for data analysis and preparation for Figs. 1–4

## Additional Information and Declarations

### Field Study Permissions

The following information was supplied relating to field study approvals (i.e., approving body and any reference numbers): The field collection permission of this study was obtained from the Wildlife Protection and Administration Office of Yunnan, China.

### Data Availability

The following information was supplied regarding data availability: The raw data has been supplied as Data S1.
